# The Elements of Materia Medica and Therapeutics

**Published:** 1852-01

**Authors:** 


*The Elements of Materia Medica and Therapeutics.* By JONATHAN PEREIRA, M. D., F. R. S., L. S. Third American edition, enlarged and improved by the author. Edited by JOSEPH CARSON, M. D., Professor of Materia Medica and Pharmacy in the University of Pennsylvania, etc. Vol. 1, pp. 338. Philadelphia: Blanchard & Lea, 1852.

We noticed at some length the third London edition of this standard work,* the first volume of which was published in 1849. The American edition, lately issued by Messrs. Blanchard & Lea, has had the benefit of the supervision of Dr. Pereira, who has corrected the London edition, with a view to its publication in the United States. The formulæ of the British colleges have been revised, and made to correspond with the last decennial editions of the London and Dublin Pharmacopœias, and the author has also, he states, "embodied all recent discoveries of importance which relate to the subjects treated of in the work." The present American edition is, therefore, substantially a *new* edition of the author's, and commends itself to favorable regard, not only from having had the advantage of his valuable supervision, but also as an example of fair and proper dealing between him and his American publishers.

We must refer our readers to our recent extended notice of the corresponding London edition of this book—which is emphatically, as the American editor justly observes, "the most comprehensive treatise on the subject in the English language."

* Vol. V., (for 1849,) p. 162.

The additions of the American editor to this volume, (which is devoted to the consideration of *inorganic bodies,*) consist chiefly in the introduction of the nomenclature, formulæ, and processes of the United States Pharmacopœia. Of his valuable notices of indigenous articles, which have enriched the former American editions, we shall have occasion to speak at length on the appearance of the second volume, the publication of which is promised "in July or August, 1852."

